# Preoperative Progressive Pneumoperitoneum in the Treatment of Hernias With Loss of Domain. Our Experience in 50 Cases

**DOI:** 10.3389/jaws.2023.11230

**Published:** 2023-04-07

**Authors:** Helena Subirana, Jaume Comas, Oriol Crusellas, Joaquim Robres, Joan Barri, Ana Domenech, Cristina Borlado, Jordi Castellví

**Affiliations:** Hospital of Sant Joan Despí Moisès Broggi, Sant Joan Despi, Spain

**Keywords:** incisional hernia, preoperative progressive pneumoperitoneum, home hospitalization, hospital at home care, large incisional hernia, abdominal wall surgery

## Abstract

**Introduction:** Surgical planning for repair of giant hernias with loss of domain needs to consider patient comorbidities, potential risks and possible postoperative complications. Some postoperative complications are related to the increase in intra-abdominal pressure caused by the reintroduction of abdominal contents into the peritoneal space. Preoperative progressive pneumoperitoneum (PPP) increases the capacity of abdominal cavity prior to hernia repair and allows for better physiological postoperative adaptation. The aim of this study is to analyze perioperative and intraoperative characteristics as well as outcomes of a cohort of patients treated with PPP prior to giant hernia repair at a single, high volume center.

**Methods:** Prospective, descriptive, observational single-center study including 50 patients undergoing PPP prior to hernia with loss of domain repair between January 2005 and June 2022. We analysed epidemiological, surgical and safety variables.

**Results:** Fifty patients were included: 43 incisional hernias, 6 inguinal hernias and 1 umbilical hernia. Mean age was 66 years (36–85). Median insufflation time was 12 days (4–20) and median insufflated volume of ambient air was 10,036 cc. There were complications during PPP in nine patients: 2 decompensation of chronic respiratory disease and 7 subcutaneous emphysema. PPP was prematurely suspended in patients with respiratory decompensation. All patients with incisional and umbilical hernias underwent open repair with mesh placement. Preperitoneal repair was performed in inguinal hernias. Three cases of hernia recurrence were reported during the follow up.

**Conclusion:** PPP is a safe and effective tool in the preoperative management of patients with giant hernias. It helps to achieve the decrease or absence of abdominal wall tension and can favour the results of complex eventroplasty techniques.

## Introduction

Patients with large hernia defects with loss of domain have a chronic and progressive weakness that changes the physiology of the abdominal wall and can affect the biomechanics of other organs (1).

Repair of these defects can lead to several pathophysiological alterations in the postoperative period related to the reintroduction of abdominal contents into a reduced abdominal cavity including abdominal compartment syndrome and acute respiratory failure secondary to an increase of intra-abdominal pressure, elevation of the diaphragm and decrease in venous return (2).

The treatment of these patients should begin in the preoperative period, aiming at achieving a progressive enlargement of the abdominal space and leading to improved tolerance to the introduction of visceral content and the reconstruction of the abdominal wall during hernia repair.

Different methods have been described to achieve primary fascial closure in patients with complex incisional hernia with loss of domain and to minimise the morbidity of abdominal wall reconstruction by reducing the risk of compartment syndrome. The most popular techniques include release of fascia: external oblique myofascial release (component separation of the external oblique aponeurosis) or release of the transversus abdominis.

Other techniques have focused on the expansion of the amount of tissue within abdominal wall. This has been achieved by the use of progressive preoperative pneumoperitoneum (PPP), tissue expanders and Botulinum toxin (3–5).

In 1940, Goñi Moreno (6) described for the first time the technique of preoperative progressive pneumoperitoneum (PPP) for the treatment of large hernias with the objective of expanding the abdominal space preoperatively and allowing for a gradual adaptation of visceral contents thereby reducing cardiorespiratory complications in the immediate postoperative period.

PPP attempts to restore part of lost abdominal cavity space to enable the reintroduction of chronically out-off domain viscera in order to avoid compartmental syndrome and allow linea alba closure.

There are no data on long-term results or randomized controlled studies of this technique because giant hernias are rare and have special characteristics. Despite being a well-known procedure, it is sporadically used and only by specialized groups (7,8).

The aim of this study is to analyze the use of PPP as a prehabilitation tool prior to repair of giant hernias with loss of domain in 50 patients.

## Methods

A descriptive, prospective, observational, single-center study was designed and developed. It was approved by hospital’s ethics committee and followed the good clinical research practice (GCP).

Inclusion criteria were patients older than 18 years with incisional hernia, umbilical hernia or groin hernia with loss of domain (Tanaka Index >25%) diagnosed by abdominal CT that underwent preoperative progressive pneumoperitoneum prior to elective surgery.

All patients meeting inclusion criteria treated at our center between 2005 and 2022 were included in the study. Patients with significant cardiorespiratory comorbidities with contraindication to PPP that did not allow to perform preoperative progressive pneumoperitoneum and active oncological patients were excluded.

Microsoft® Access 2007 database and SPSS v16 programs were used for data collection and statistical analysis.

30 of the 50 cases were also included in the Registro Nacional de Eventraciones (EVEREG), a database led by the Abdominal Wall Surgery section of the Asociación Española de Cirujanos (AEC) with the objective to study the implementation of surgical techniques, clinical characteristics and results of surgical treatment.

Demographic and clinical variables studied were: age, gender, body mass index (BMI), hernia type (incisional, groin or umbilical) and history of previous hernia repair.

The following treatment related variables were collected: duration of PPP (days), volume of insufflated air, days of preoperative hospital or home hospitalization unit (HHU) admission, complications during PPP treatment, measurements of hernia defect (using EHS classification), hernia repair surgical technique, placement of prosthetic material, postoperative complications, postoperative length of hospital stay and hernia recurrence during follow-up.

All patients were informed of the intervention and the procedure, authorizing them with the signature of the informed consent.

### Technique

Patients selected for PPP prior to elective hernia repair were referred for pre-anesthetic evaluation and scheduled for hospital admission 1–3 weeks prior to surgery.

Patients underwent CT-guided catheter placement in the interventional radiology suite. The catheter was placed at a point away from the hernia sac, always taking into account previous incisions and looking for adhesion-free locations. For patients who could not undergo CT guided catheter placement, the procedure was performed in the operating room under local anesthesia and sedation, by initially creating a small pneumoperitoneum with a Veress needle to place the intraperitoneal catheter.

Following successful catheter placement, 500 cc of ambient air is slowly administered into the peritoneal cavity during the first post-puncture day. If well tolerated, daily insufflations are performed either at home, under the care of a HHU or as an inpatient. An average of 4–5 sessions per week are performed by trained nurses, administrating between 500–1,000 cc of air per session and monitoring tolerance. Insufflation is suspended in case of pain, tachycardia, hypertension or hypotension, or desaturation. Total volume at the end of the insufflation varies depending on the size of the defect and the adaptability of each patient, ranging between 5 and 16 L. This volume is usually reached in 2–3 weeks.

Follow-up is performed mainly by clinical examination of the abdominal wall, evaluating the tension of the musculature in the lateral parts of the abdomen. The optimal timing of hernia repair is when the lateral muscles of the abdomen are completely distended.

Patients are instructed to perform respiratory physiotherapy exercises, stop smoking and employ skin hygiene measures during PPP therapy.

Once optimal abdominal muscle distention is achieved, surgical repair is carried out using standard hernia repair techniques with mesh according to each patient’s type of deffect.

Postoperative follow-up was at 30 days, 6 months, 1 year and 2 years after surgery with clinical exam. Radiographic control is performed with an abdominal CT only if it is necessary in case of suspected hernia recurrence or complications like seroma or hematoma, but not systematically.

## Results

### Patient Demographics and Hernia Characteristics

From 2005 until 2021, 50 patients met inclusion criteria and were included in the study. There were 24 men and 26 women with a mean age of 66 years (range 36–85). The most frequent comorbidity was obesity, with an average BMI of 34 (range 24–50). Other common comorbidities were chronic obstructive pulmonary disease, diabetes and high blood pressure.

The majority of patients (43) had incisional hernias, 6 had inguinal hernias and 1 patient had an umbilical hernia. All patients had large defects with associated loss of domain. The average size of the hernia defect was 15 cm length × 20 width (W3 of EHS classification (range 4–35/5–35).

18 of the 50 cases (36%), had recurrent hernia: most of them had original repair with mesh while 2 patients (11%) had hernia repair without mesh (Table 1).

**TABLE 1:** Epidemiological variables.

Characteristics	PPP n: 50
Mean age	66 (36–85)
Sex	
Male	24 (48%)
Female	26 (52%)
BMI	34 (24–50)
Type of hernia	
Incisional hernia	43 (86%)
Umbilical hernia	1 (2%)
Groin hernia	6 (12%)
Previous hernia repaired	18 (36%)
With mesh	16 (88%)
Without mesh	2 (11%)

### Preoperative Progressive Pneumoperitoneum

84% of patients (42/50) had successful placement of the peritoneal catheter under CT guidance. The remaining 8 patients had catheter placement in the operating room.

In 15 patients (30%) progressive preoperative pneumoperitoneum was performed by HHU and in 35 (70%) the patient was admitted to hospital (Table 2).

**TABLE 2:** Surgical variables.

Characteristics	PPP n: 50
Median diameter of hernia repair (cm)	15 × 20 cm (4–35/5–35)
Insufflation time	12 days (4–20)
Mean insufflated volume of air (cc)	10,036 cc (4,080–15,900)
Hospitalization during PPP days	
Conventional hospitalization	35 (70%)
Domiciliary hospitalization	15 (30%)
Abdominal Wall repair technique	
Anterior CST	40
Preperitoneal approach (inguinal hernia)	6
Primary closure and onlay mesh	4
Mean hospital stay after surgery	19 days (6–70)
Recurrence	3 (6.8%)

Mean insufflation time was 12 days (range 4–20) and mean total volume of insufflated ambient air was 10,036 cc (range 4,080–15,900).

Adverse events occurred in 9 patients during PPP: 2 patients developed acute exacerbations of their underlying chronic respiratory disease and 7 patients developed subcutaneous emphysema. All observed adverse events were Grade I or II by the Clavien-Dindo classification. There were no Grade III or higher adverse events during PPP. In patients with respiratory decompensation, PPP was suspended. Both patients underwent successful PPP therapy several months later without incidences. Subcutaneous emphysema disappeared spontaneously in all patients over a period of several weeks (Table 3).

**TABLE 3:** Complications associated with the PPP and surgical repair according to Clavien-Dindo Classification.

	Associated with PPP	Associated with repair
Total	9 (18%)	30 (60%)
Grade I	Subcutaneous emphysema 7	Seroma 7
	Respiratory decompensation 2	Wound infection 3
		Wound necrosis/dehiscence 8
		Adynamic ileus 4
Grade II		Pneumonia 2
		Urinary infection 2
		TEP 2
Grade III		Intestinal perforation 1
Grade IV		Compartment syndrome 1

### Hernia Repair Surgery and Outcomes

All patients with incisional or umbilical hernia underwent open hernia repair with mesh placement. In 40 of the 44 incisional and umbilical hernias, modified anterior component separation (SAC) technique described by Carbonell and Bonafé (9) was used.

In 13 cases we had to use a sandwich technique with double mesh placement because a primary closure was not possible to develop despite SAC.

In 4 of the 44 cases the mesh was placed in an onlay position, with a primary fascial closure.

Preperitoneal repair was used for inguinal hernia repair.

Mean postoperative hospital stay was 19 days, with a range between 6 and 70 days.

Regarding medical complications during the postoperative period while patients were admitted, 2 patients developed pneumonia, 2 developed urinary tract infection and 2 patients developed pulmonary embolism in the postoperative period despite receiving low-molecular weight-heparin. There was a case of unrecognized intestinal perforation during hernia repair that required surgical reintervention with good outcome. One of the 50 patients died after repair of giant inguinoscrotal hernia due to postoperative severe respiratory insufficiency and compartment syndrome evolving to multiorgan failure (Table 3).

Eighteen patients (36%) presented skin complications: 7 patients developed a seroma, 8 had skin necrosis and 3 patients developed wound infections that were treated with antibiotics.

During follow-up, 6 patients developed cutaneous dehiscence of the surgical wound that was treated with negative pressure therapy with good results. At 12 months after surgery, 3 patients (6%) had recurrence of their incisional hernias.

## Discussion

The high complexity of patients with large abdominal wall hernias lead the surgeons to have a better knowledge of organs and systems affected by an absence of a functional abdominal cavity (10).

Progressive preoperative pneumoperitoneum technique has improved the treatment of patients with hernias with loss of domain since the abdominal cavity can be expanded in order to maintain the herniated volume in place and improve respiratory adaptation, so that the repair can be carried out with appropiate safety and the risk of abdominal compartment syndrome can be reduced (11).

Although its use has not been fully widespread in most hospitals, teams of specialized abdominal wall surgeons who have incorporated this technique in the treatment of complex abdominal wall diseases have reported good results with an acceptable risk (12).

One of the main controversies is defining which patients will benefit from this technique. Patients with loss of domain are considered tributaries of preoperative progressive pneumoperitoneum but there is no consensus in the literature on the definition of hernias with loss of domain or the amount of gas that should be insufflated in each patient (13).

One of the most accepted definitions of hernias with loss of domain is when the hernia sac contains more than 20% of the abdominal content or when the hernia defect is larger than 10 cm in transverse diameter (14).

Tanaka et al. (15) described a technique for calculating the hernia sac volume and the abdominal cavity volume based on abdominal computerized thomography that eliminates the need for subjective criteria for inclusion in a PPP program and predicts the amount of gas that must be insufflated into the abdominal cavity.

This group suggests that the PPP only has to be performed if the volume ratio HSV/ACV is more than 25%.

Our group include patients with huge incisional hernias with >25% of abdominal visceral content herniated, although the main inclusive criteria is based on clinical examination.

In our experience CT can underestimate, in some cases, the real volume of the hernia sac content because of the patient position.

Dumont et al. described that one of the additional effects of pneumoperitoneum is that increases the length of the rectus abdominis muscles, which could be determined by topographic measurements by CT scan (16). They report that this increase could facilitate fascial repair in large hernias and eventrations, with minimal tension closures.

In our opinion it is not necessary to carry out a radiographic control with CT sistematically although it would be interesting to know the amount of increase in muscles length.

Follow up with clinical examination is enough to evaluate musculature tension.

Abdomen CT should be done only in selected cases or in complications.

Some studies use CO2 and oxygen to perform PPP but it is known to have an absorption 4 times faster in the intraperitoneal space than the ambient air (17). In all our patients ambient air is administered.

No complications have been reported with ambient air insufflation. The expected complications could be bacterial peritonitis or intraabdominal abscesses but we did not find these in our study.

To control the volume of insufflation, some authors perform intra-abdominal pressure measurements. In our case, we place a daily volume of air that can vary between 0.9 and 1 L, depending on the patient’s tolerance and evaluating respiratory difficulty, pain or nausea, which correlates directly with the increase in abdominal pressure.

Several authors consider that, after monitoring the abdominal circumference and respiratory function of the patient, there is no benefit of maintaining pneumoperitoneum beyond 6–10 days of insufflation (18).

In our experience, the average insufflation time was 12 days, with individual variability depending on the tolerance of each patient.

Moreover, we think that PPP allows muscular relaxation by progressive distention of the abdominal wall musculature that is retracted and also acts as the pneumoperitoneum performed in laparoscopic surgery, facilitating the dissection of adhesions in a non traumatic way.

In addition, it has been described that from the second week it stimulates the immune system and improves the macrophage’s cellular response, favoring subsequent wound healing (2).

Most studies only describe the use of progressive pneumoperitoneum for large incisional hernias repair. In our group we also use it to solve giant inguinal and umbilical hernias, with good results.

Coehlo et al. (19) used the pneumoperitoneum to allow the adaptation of the abdominal wall and avoid the placement of prosthetic material in 30 of the 36 patients in whom they used PPP. Currently, use of meshes has become widespread, especially in complex abdominal walls. In all our patients placement of prosthetic material was performed.

Recurrence rate of incisional hernia after simple closure ranges between 7% and 24%. We believe that it is necessary to associate prosthetic material in hernia repair with preoperative pneumoperitoneum.

Complications described related to this technique are mainly local. They can appear at puncture time or during the pneumoperitoneum maintenance. Those related to puncture are perforations, intraluminal placement in the GI tract or bleeding. Those related to maintenance are shoulder pain, GE reflux, satiety, respiratory distress, subcutaneous emphysema. In these situations the frequency and amount of insufflated air should be reduced. Other complications include bleeding caused by traumatic lysis adhesions, pneumatic dissection of the gallbladder bed, splenic rupture or puncture site infection (20). In our series we have shown mild complications, most of them subcutaneous emphysema and in 2 cases respiratory decompensation that could be solved without incidences.

The low incidence of serious complications arising from the progressive pneumoperitoneum, all of them less than grade II in Clavien Dindo classification (21), and the long hospital stay to complete the procedure, made us consider to perform it by the Home Hospitalization Unit.

Home Hospitalization is an effective and efficient healthcare modality in medical and surgical pathology in which, for a limited time, health practitioners provide active treatment at the patient’s home (22,23).

Only 27% of the patients in our series benefited from the HHU regime because we started including patients on it in 2016, obtaining the same final results, with a good acceptance by the patient and showing a decrease in health costs.

Bueno Lledó’s group developed a study in which they showed the results of combining preoperative progressive pneumoperitoneum with botulinum toxin infiltration for the preparation of patients with giant hernias with loss of domain (11). They propose that the administration of botulinum toxin helps to reduce lateral tension forces on the hernia defect and increases the elongation of the lateral muscles of the abdomen with a subsequent increase of the volume of the abdominal cavity, which will allow a tension-free abdominal reconstruction with PPP.

Usually botulinum toxin infiltration is used in smaller hernias as a single technique to achieve a better and easier closure. We think it can be helpful in reducing muscular tension and the combination of these two techniques can help us to achieve better results, allowing a wall abdominal closure without tension and a decrease of recurrent incisional hernias but more studies are needed.

Although there is no consensus in the literature for the treatment of patients with complex abdominal wall disease, the published series in recent years, suggest that PPP can be used safely, providing better surgical results than primary repair techniques.

Finally, other factors that influence and can provide better results are respiratory physiotherapy, stop smoking, weight loss and hygienic skin care in these patients (24).

## Conclusion

Surgical repair of giant abdominal wall defects requires a specific preoperative preparation with hygienic-dietary measures and techniques that increase thoracoabdominal capacity. We demonstrate that progressive preoperative pneumoperitoneum is a safe and easy technique, which can complement complex eventroplasty techniques such as component separation, providing advantages in the optimization of patients with large abdominal wall defects and obtaining good results, regarding the operative technique and the multisystemic adaptation of the patient.

Catheter placement procedures and pneumoperitoneum maintenance are well tolerated by patients and with few associated complications. They are also easily reproducible in specialized centers, with abdominal wall surgeons and intensive care unit.

## Data Availability

The original contributions presented in the study are included in the article/supplementary material, further inquiries can be directed to the corresponding author.
